# Ethics Standards (HRPP) and Public Partnership (PARTAKE) to Address Clinical Research Concerns in India: Moving Toward Ethical, Responsible, Culturally Sensitive, and Community-Engaging Clinical Research

**Published:** 2014-09-07

**Authors:** Tal Burt, Yogendra K Gupta, Nalin Mehta, Nagendra Swamy, Vishwas Sovani, Marjorie A Speers

**Affiliations:** 1 Duke Clinical Research Unit & Duke Clinical Research Institute, Durham, NC, USA; 2 All India Institute of Medical Sciences, New Delhi, India; 3 Manipal Health Enterprises, Karnataka, India; 4 Revogenex Inc., Duluth, GA, USA; 5 Association for the Accreditation of Human Research Protection Programs, Washington, DC, USA

**Keywords:** Ethics standards, Public awareness, Clinical research, India

## Abstract

Like other emerging economies, India’s quest for independent, evidence-based, and affordable healthcare has led to robust and promising growth in the clinical research sector, with a compound annual growth rate (CAGR) of 20.4% between 2005 and 2010. However, while the fundamental drivers and strengths are still strong, the past few years witnessed a declining trend (CAGR −16.7%) amid regulatory concerns, activist protests, and sponsor departure. And although India accounts for 17.5% of the world’s population, it currently conducts only 1% of clinical trials.

Indian and international experts and public stakeholders gathered for a 2-day conference in June 2013 in New Delhi to discuss the challenges facing clinical research in India and to explore solutions. The main themes discussed were ethical standards, regulatory oversight, and partnerships with public stakeholders. The meeting was a collaboration of AAHRPP (Association for the Accreditation of Human Research Protection Programs)—aimed at establishing responsible and ethical clinical research standards—and PARTAKE (Public Awareness of Research for Therapeutic Advancements through Knowledge and Empowerment)—aimed at informing and engaging the public in clinical research.

The present article covers recent clinical research developments in India as well as associated expectations, challenges, and suggestions for future directions. AAHRPP and PARTAKE provide etiologically based solutions to protect, inform, and engage the public and medical research sponsors.

## Introduction

In June 2013, a group of local and international clinical research and public stakeholders gathered in New Delhi to tackle a unique challenge: the fortunes and prospects of clinical research in India, one of the most promising global clinical research environments, appeared to be in decline. Conference stakeholders included experts from industry, academia, regulatory bodies, and the private sector as well as representatives from patient advocacy and public activist groups, non-governmental organizations (NGO’s), and the media. The scope of the representation was a reflection of the organizers’ collective belief that medical research in general and clinical research in particular require collaboration of both professional and non-professional segments of society, a rare undertaking in usual practice, to understand the challenges facing the sector and to identify effective solutions.

## Background

### Clinical research in India

#### Motivation for global and Indian clinical research

India has powerful drivers and attractive capabilities favoring clinical research. Rapid increase in life expectancy along with increases in the prevalence and burden of chronic illness are not yet matched by the provisions of the healthcare system or indigenous medical research capabilities [1–3]. The aspirations of the rapidly emerging economy, on track to become the world’s second-largest by 2050, indeed include becoming self-sufficient in providing care to its growing population and basing such care on home-grown, evidence-based research [4]. Ethnic differences in the genetically heterogeneous population may affect presentation and outcomes of interventions, making generalizations from studies done elsewhere difficult and providing a strong case for indigenous clinical research [2,5–8].

**Figure 1:**
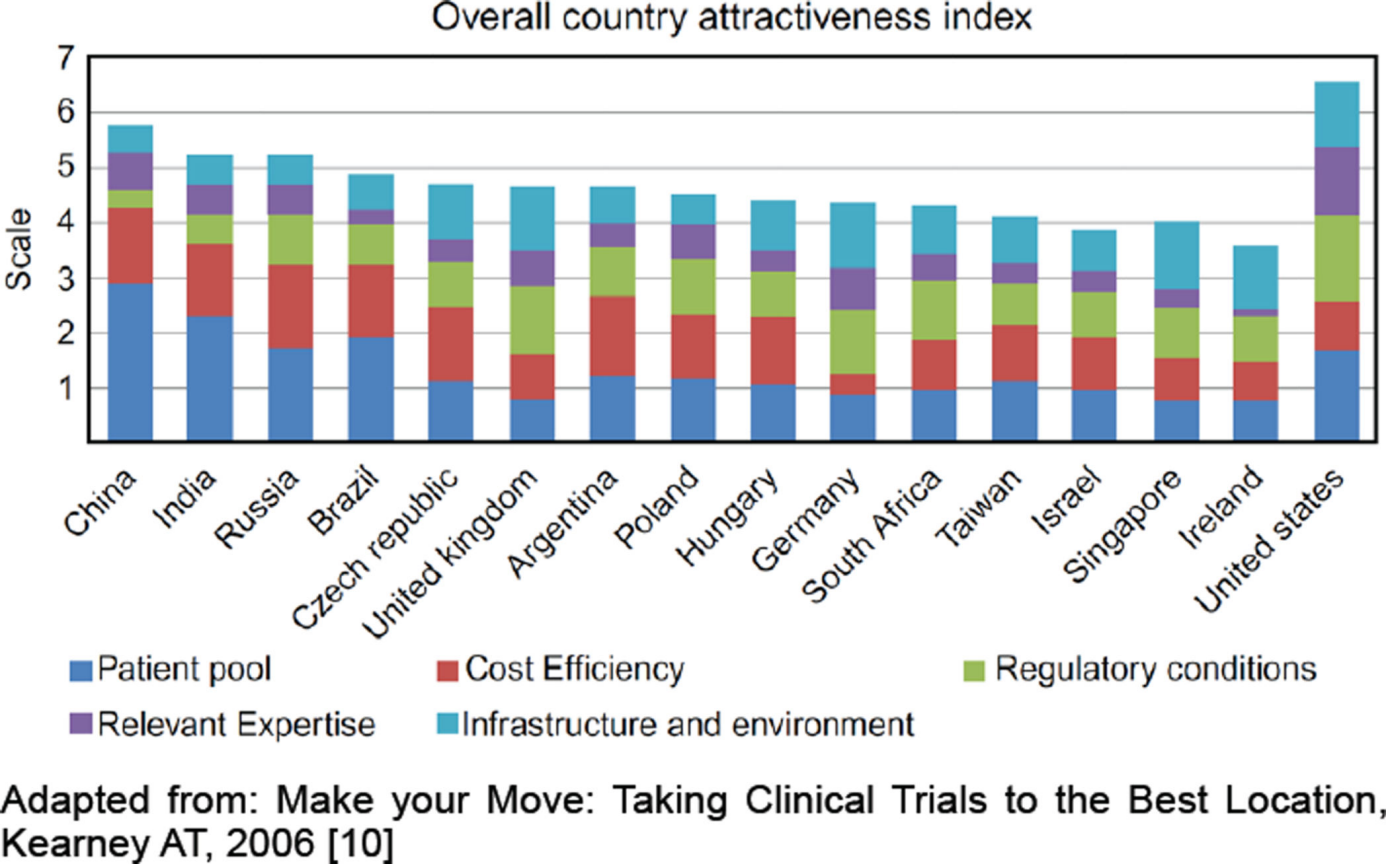
Most Attractive Global Locations for the Conduct of all Clinical Trials Outside the United States: 2006.

**Figure 2:**
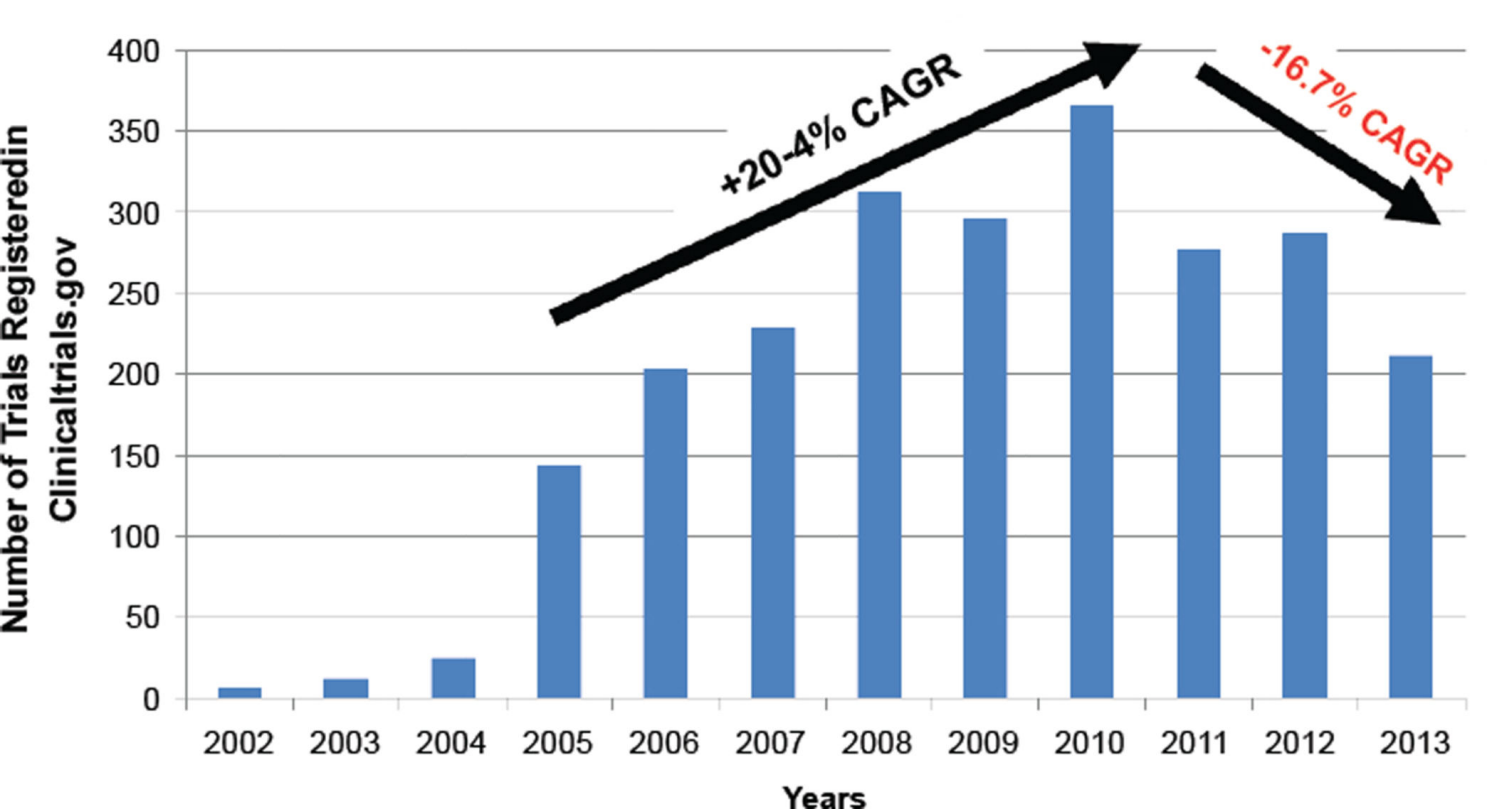
ClinicalTrials.gov. All India Clinical Trials 2002–2013 Data were obtained from ClinicalTrial.gov on January 9, 2014. Methods: “Advanced Search” option was used. “India” entered in “Country” field. “First Received” field was used to include dates “From 01/01/…. To 12/31/….” for each year from 2002 through 2013. Compound annual growth rate (CAGR) was used for the periods 2005–2010 and 2010–2013 (years prior to 2005 were deemed to contain data that were not meaningful) using the formula:
$$CAGR(t_{0},t_{n})={\left(\frac{V(t_{n})}{V(t_{0})}\right)}^{\frac{1}{t_{n}-t_{0}}}-1;$$
*V* (*t*_0_): *Start Value*; *V* (*t_n_*): *Finish Value*; *t_n_* − *t*_0_ : *Number of Years*

India offers many advantages likely to appeal to medical researchers: well-trained (including many returning, Western-trained) physicians and investigators, an English-speaking environment, treatment-naïve populations, large healthcare center catchment areas, access to world-class information technologies and data management infrastructure, competitive operational costs (40–60% reduction when compared with Western sites), and sustained economic growth [9]. A study in 2006 by Kearney found India second only to China on the Country Attractiveness Index for Clinical Trials (primarily on the basis of patient pool and cost efficiency); however, India was less attractive than most countries in terms of regulatory conditions, infrastructure, and environmental factors (Figure 1) [10]. Advantages also include a harmonized regulatory system with an auditable clinical trial registry [11]. In addition, collaboration with Western universities and research networks has contributed to the establishment of high clinical research standards [12,13]. Notwithstanding the considerable potential and strengths, and after a 2003 regulatory overhaul (Schedule Y [14]) initially translating into robust growth (2005–2010), the clinical research sector has witnessed a decline in recent years (Figure 2) [15–19]. While India represents 17.5% of the world’s population, it has conducted only 1.75% of the world’s clinical trials (2749 of 157,327) and currently only conducts 1.08% of global trials (211 of 19,599 trials registered with ClinicalTrials.gov from January 1 to December 31, 2013) [20–21].

### India’s regulatory environment

The Indian clinical research environment has come under increased scrutiny by public activists and the media, and subsequently by the Indian Supreme Court for perceived unethical practices in the conduct of clinical trials (Table 1) [16,24,26,30,32,36–39]. A report by the Indian Parliamentary Standing Committee identified an understaffed and under-resourced Central Drugs Standard Control Organization (CDSCO), including deficiencies in enforcing regulations, “collusion” with industry sponsors, and claims of exploitation of Indian citizens by foreign pharmaceutical companies [40]. Lack of regulatory clarity and lengthy turnaround times for clinical trial approvals have deterred both local and foreign sponsors from conducting clinical trials in India and may have contributed to the reduction in the number of clinical trials in India since 2010 (Figure 2) [18,39]. On January 30, 2013, the Ministry of Health and Family Welfare of India issued an amendment to the Drugs and Cosmetics Act of 1940 and the Ministry of Law issued a set of guidelines on patient compensation that included the need to pay should an experimental drug fail to show the intended therapeutic benefit (including in cases where research participants are allocated to placebo interventions) (Table 2) [25,30], Human Research Protection Program (HRPP) Conference, June 29–30, 2013, New Delhi, India].

In total, 169 participants, including 39 speakers, representing all clinical research stakeholders gathered for the 2-day conference. Fourteen participants were from the United States, the United Kingdom, Taiwan, and Saudi Arabia, and the rest were from India. The various attendees represented industry (56) healthcare (48), academia (40), NGO, public and patient advocacy (16), and regulatory (9) stakeholders.

### Understanding the challenges and their causes

On day 1, conference participants viewed interactive and targeted presentations; on day 2, the group participated in focused discussions aimed at identifying and understanding the causes of challenges and proposing tangible solutions. Three factors and respective deficiencies emerged as probable causes of the observed difficulties facing clinical research in India:

#### Ethical standards of clinical research

Recent widespread reports of violations suggest that research standards, especially those pertaining to the protection of human participants, may not be properly enforced (Tables 1 and 2)[19,22,23,26,33,34,36,38,40,41,48–50]. The responsibility of ensuring respect for and protection of study participants is the foundation of the partnership between the researcher and the human research participant. Several principles of importance have been identified as essential to the ethical conduct of clinical trials: superiority of benefit over risk (beneficence), fairness in assignment and access (justice), and validity and freedom of bias (integrity) [51]. However, it is autonomy and respect, manifested in the adequacy of the informed-consent process and compensation for adverse outcomes, that appear to dominate the concerns of stakeholders [26,33,38,47].

#### Regulatory process

The regulatory system that governs clinical research in India has undergone significant revisions and acquired meaningful strengths in the first decade of the new millennium, including harmonization of global good clinical practice (GCP) regulations and the establishment of mandatory clinical trial registration [9,11,14]. In recent years, however, there have been numerous incidences of regulators having difficulty enforcing regulations due to inadequate staffing and resources, claims of industry bias, and lack of clarity and transparency (Tables 1 and 2) [39,40]. Criticism pointed to a lack of equitable and feasible regulations (e.g., determination of serious-adverse-event causality) that take into account sociocultural characteristics, diversity, and vulnerabilities [30,38,47]. There is also a need to ensure the quality of research training and conduct as well as compliance of investigators and research operators [13].

#### Public perceptions and engagement in clinical research

This encompasses the following:

Existence of cultural divides (professional, national, international) [12,16,24,49,52]

Impaired public awareness, comprehension, empowerment, engagement, and partnership in clinical research, with a substantial and an active portion of the population (15–25%) holding negative perceptions of clinical research and being distrustful of researchers and regulators [16].

Lack of transparency and impaired communication amongst professional and public stakeholders limits reconciliation of different perspectives and the ability to establish consensus, working relationships, and collaborations [53–55].

With holding important contributions that the public may provide as an active partner in the clinical research process (communicating therapeutic preferences; enhancing study design; helping with data collection; enforcing standards; and helping with research funding, lobbying, and public policy [53,54,56,57]).

While available data are not sufficient to establish causality, these recent challenges have been associated with a decline (−16.7% compound annual growth rate [CAGR]) in the number of clinical trials conducted in the 2010–2013 period after a promising growth of 20.4% in the preceding 5 years (Figure 2).

### Etiologically based solutions

Regulatory efforts together with the establishment of HRPPs and public awareness programs (e.g., PARTAKE [Public Awareness of Research for Therapeutic Advancements through Knowledge and Empowerment]) complement each other’s scope and strengths and offer comprehensive, synergistic solutions to the complex problems facing clinical research in India.

#### Regulatory review and overhaul

Regulatory reorganization will ensure availability of resources and expertise to allow enforcement of regulations through education, training, accreditation, monitoring, and auditing of investigators and sites. Regulators should establish transparency and clarity regarding guidelines and responsibilities of clinical research professionals, and they should eliminate bias and conflicts of interest. The hoped outcomes of these actions are ensuring human research protection, verifying the quality of research applications and study operations, increasing trust in the regulatory system, reducing application turnaround times, and encouraging engagement and partnership by all stakeholders in clinical research. In the aggregate, these are also expected to lead to the generation of high-quality and credible research data.

Amendments proposed by regulators initially included provisions that were considered either not feasible or inconsistent with sound scientific methodology (e.g., compensation of all adverse events in clinical trials, expectation of therapeutic effect, attribution of placebo assignment to adverse events, expectation of ethics committees to determine compensation), yet these were decried by activists as containing insufficient protections for research participants [19,30,38,39]. By the end of 2013, the Chaudhury Expert Committee submitted recommendations that addressed earlier concerns [58].

ICMR (Indian Council of Medical Research), the national apex body to monitor clinical research in India is in the process of addressing clinical research challenges in India through collaboration with WHO (World Health Organization), Forum for Ethical Review Committees in the Asian & Western Pacific Region (FERCAP) and Strategic Initiative for Developing Capacity in Ethical Review (SIDCER).

#### HRPP initiatives

HRPP initiatives are a component of global AAHRPP (Association for the Accreditation of Human Research Protection Programs) efforts to promote responsible and ethical clinical research [59]. AAHRPP’s mission is to protect human participants and enhance research quality through an accreditation process. The process aims to establish high, harmonized, and consistent standards of research operations, including standards for investigators, study teams, institutions, and ethics committees involved in clinical research. The process starts with self-assessment of compliance with a set of objective standards followed by onsite evaluation and council review by AAHRPP staff. The process emphasizes ongoing education and training, continuous process improvement, performance-based objective standards, and repeat accreditation. A robust informed-consent process (e.g., audio-video recording), monitoring, and auditing are examples of high standards that are involved. As of December 2013, India has 3 accredited institutions, China has 2, Singapore 1, South Korea 6, Taiwan 1, Mexico 1, and several additional institutions from emerging economies are in the process of seeking AAHRPP accreditation [60].

AAHRPP standards are grouped into 3 domains (Table 3): I) organization; II) institutional review board or ethics committee; and III) researcher and research staff. Each standard is further divided into elements that provide additional details on specific accreditation requirements [60].

#### PARTAKE

The PARTAKE initiative started in India and then expanded to the United States and China, where surveys are underway [16]. Public awareness, perceptions, and consequent attitudes toward clinical research may impact regulatory policies, guide research priorities, and shape growth in the sector; however, distrust, lack of awareness, and misconceptions of clinical research have been identified as key barriers to participation in clinical trials [61–65]. The PARTAKE initiative is based on the premise that an informed, participating public is invaluable for the following ethical, methodological, and operational reasons:

##### A. Ethical

These can be divided into rights and obligations.

Rights: Participants in clinical research have the right to make informed decisions about participation in research [49,66,67] and are better positioned to protect their rights when they are knowledgeable of clinical research [68,69].

##### Obligations

Societies who desire and demand advanced therapeutics and individuals who are willing recipients of innovative treatments have an implied obligation to be part of the process that develops and approves them (i.e., becoming active participants, partners, and contributors to the process [53,54,67]).

##### Methodological

A wide and representative sample of participants in clinical research is essential to ensure adequate generalization of the findings to the population at large [57,70]. Informed persons and research participants could assist in enforcement of research standards (ethical and methodological) and increase the quality of data generated.

##### Operational

A key obstacle to medical progress is the limited participation in clinical research. This makes research more costly and less powerful in detecting meaningful therapeutic effects, and it delays the arrival of new treatments to those who need them [57,65,71–73].

The PARTAKE program includes 7 steps (Table 4), of which the first 2 have been initiated. Results of a pilot survey (albeit being limited to 1 Indian metropolitan [16]) suggest that the Indian public is aware of some key features of clinical research (e.g., purpose, value, voluntary nature of participation) and supports clinical research in general but is unaware of other key features (e.g., compensation, confidentiality, protection of human participants) and exhibits some distrust in the conduct and reporting of clinical trials [16]. Challenges facing the initiative and proposed solutions are summarized in Table 5.

## Conclusions and Future Directions

The clinical research environment in India is challenged by a complex interplay of sociocultural, regulatory, ethical, economic, and scientific factors likely representing similar dynamics in other emerging economies. In a meeting of public, activist, media, government, academia, and clinical research industry stakeholders, a comprehensive effort was undertaken to examine the sources of the difficulties and propose solutions. The principles and direction of AAHRPP (participant protection) and PARTAKE (public awareness) programs were identified, together with a regulatory overhaul, as complementary, synergistic, and crucial to any meaningful and lasting solutions. Etiologically based proposed strategies were focused on improving regulatory oversight, establishing research participant protection programs, and enhancing public awareness, empowerment, and engagement in clinical research. Ensuring a robust informed-consent process, promoting investigator education, establishing clarity and transparency of regulations, establishing community advocates, and creating a research participant bill of rights were some of the activities proposed by conference participants. These solutions should be carried out in a manner that is cognizant and respectful of sociocultural customs, diversity, and vulnerabilities of the population. These insights may be relevant to other emerging economies like China and Brazil, which (like India) have experienced a rapid introduction of modern research, and are looking to establish indigenous clinical research and bring the promise of affordable medical research and healthcare to their people.

## Figures and Tables

**Table 1:** Examples of Public Perception of Clinical Research in India.

Research Element	Perceptions	Source	Potential Implications/Consequences
	**Negative**		
**Human participation**	“Human guinea pigs”	Allahabad High Court [22]; PARTAKE survey [16]	Indians are exploited by foreign and rich industry entities for financial gain [23].
	Exploitation of vulnerable populations (illiterate or impoverished) [24]	Petition of Supreme Court [25]	Clinical trial applications put “on hold” amid revision of the regulatory system [19]
**Adverse events**	Violation of “right to live” under the constitution	Allahabad High Court [22]	Proposal to prosecute trial-related adverse events under the Indian Penal Code
	Unnecessary and exploitative	Media [26]; activists; surveys [27]; government compensation committee [28]	Adverse events are always due to trial participation and therefore should be compensated for; hold on clinical trials; withdrawal of drugs with favorable benefit/risk ratios [29]
**Placebo assignment**	Violation of the right to effective treatments	Government compensation scheme [30]	Proposal to compensate study participants for lack of therapeutic effect [30]
**Informed consent**	The process is not “informed” and “consent” cannot be assumed; consequence of paternalistic model of health care [31]	National Human Rights Commission [32], media [33,34], activists, PARTAKE survey [16]	Addition of audio-visual recording to informed-consent process [35]
	**Positive**		
**Medical knowledge**	Enhancing public health	Surveys [16,27]	Large majority endorses research
**Human participation**	Voluntarism	Surveys [16,27]	Majority interested in participating in research
	Altruism	Surveys [16,27]	Majority endorses altruism as the only valid motivation to participate in research

PARTAKE, Public Awareness of Research for Therapeutic Advancements through Knowledge and Empowerment.

**Table 2:** Events Related to Standards of Clinical Research in India.

Event	Description	Impact	Notes
**Deaths in HPV vaccine study**	4 teenage girls die during vaccine trial [41]	Foreign sponsors (Gates Foundation, PATH) blamed for using Indians as “guinea pigs” [41]	A government-commissioned committee confirmed the deaths were not related to the vaccine [41]
**CDSCO report**	Government report finds deficiency in enforcement of regulation [40]	Activist protests, Supreme Court intervention, regulatory overhaul, hold on clinical trial approvals [18,19,30]	“Collusion” of regulators with industry sponsors is decried in the media; it is inferred by the identification of copycat letters sent by physician experts to the regulators in support of pharmaceutical clinical trial applications
**NGO petitions**	Requesting inquiry into the small number of trial adverse events and deaths resulting in compensation [33]	Leading to Supreme Court January 2013 decision (see below)	
**Supreme Court decision**	Petitioned by activists to investigate compensation for trial-related adverse events and deaths	Regulatory overhaul; 3-month hold on clinical trial application approval [19,25]	
**NIH trials in India “on hold”**	June 2013 announcement amid perceived regulatory and legal uncertainties [42]	Reduction in the amount of clinical research [19,43]	
**FDA inspections**	FDA issues warning letters to Ranbaxy, Wockhardt, and Jubilant	Diminished confidence in Indian pharmaceutical industry	Quotes from FDA inspectors: · “submitted untrue statements of material fact” [44] · “concerns about integrity of all data” [45] · “innocent ignorance, surprising sloppiness, malicious malfeasance” (HRPP conference, 2013)
**Quintiles closing early-phase unit**	Collaboration with Apollo Hospitals, Hyderabad–opened in 2010	One of only 2 early-phase units with international collaboration	Closed due to “challenging external business environment” [46]
**Compensation schemes**	Key feature of activist and judicial demands: compensation for adverse events and lack of therapeutic effect; ethics committees responsible for determining liability in clinical trials	Initial compensation proposals prompt sponsor and investigator reluctance to conduct trials [18]	Unresolved issues: determination of adverse-event causality; expectation of efficacy in clinical research; liability of placebo assignment; ethics committee resources and expertise to determine liability in clinical trials [38,30,47]

FDA, U.S. Food and Drug Administration; HPV, human papillomavirus; HRPP, human research protection program; NGO, non-governmental organization; NIH, U.S. National Institutes

**Table 3:** AAHRPP Domains and Standards.

Domain	Standards
**Domain I: Organization**	I-1: Organization has systemic and comprehensive human protection program
	I-2: Adequate resources exist for program implementation
	I-3: Transnational research activities are consistent across sites and respectful of local laws and cultural context
	I-4: There is adequate response to research participant concerns
	I-5: There are performance metrics and continuous process improvement
	I-6: Financial conflicts of interest are identified and managed to minimize impact
	I-7: There are policies and procedures governing use of investigational products
	I-8: There is collaboration with public, industry, and public stakeholders
**Domain II: IRB or EC**	II-1: Structure and composition of IRB/EC is appropriate to the amount and nature of the research reviewed
	II-2: IRB/EC evaluates each research protocol to ensure protection of participants
	II-3: Approved protocols abide by applicable laws and regulations
	II-4: Additional protection is provided to vulnerable research participants
	II-5: IRB/EC maintains documentation of its activities
**Domain III: Researcher and research staff**	III-1: Adherence to ethical principles and rights and welfare of research participants are primary concerns when designing study
	III-2: Researchers and staff meet all regulatory requirements and applicable laws

Adapted from: aahrpp.org [60] EC, ethics committee; IRB, institutional review board.

**Table 4:** PARTAKE Program Steps.

	Steps	Description
**Step 1**	PARTAKE survey of knowledge and perceptions of clinical research	Public survey to assess knowledge and perceptions of clinical research and inform education, public awareness, and participant protection programs
**Step 2**	Stakeholder collaborations	Collaborations with industry, health care providers, academia, regulatory, patient advocacy groups, media, and the public at large [68]
**Step 3**	Development of awareness and engagement programs	Educational programs will be created to address the knowledge and awareness gaps identified in the survey
**Step 4**	Research on PARTAKE impact	Research on impact of PARTAKE educational programs on public knowledge and awareness of clinical research
**Step 5**	Research on PARTAKE impact on clinical research	Research on impact of changing public knowledge, awareness, and attitudes on clinical research
**Step 6**	Enhancing clinical research programs	Development of “participant protection” and “public-friendly” clinical research programs
**Step 7**	Development of an endorsement and rating/scoring program	Establishing a rating/scoring program that includes representatives of all stakeholders involved in clinical research–to grade research operations for their “participant protection” and “public-friendly” properties and establish a process for endorsement and improvement of research operations

PARTAKE, Public Awareness of Research for Therapeutic Advancements through Knowledge and Empowerment.

**Table 5:** Challenges Facing PARTAKE Initiative and Proposed Solutions.

Challenges	Solutions
Lack of public trust in investigators, sponsors, and regulators	Engage the public/community; invite patients/public to participate in clinical research decision-making; empower, partner, and establish transparency in clinical research operations; establish research participant bill of rights
Fragmentation of the research environment	Establish a comprehensive public relations strategy; improve communication amongst stakeholders; use common language; engage the media; hold “open-house” activities; engage community advocates
Myths and misconceptions	Increase awareness, educate, and reduce stigma; establish community advocates (members of the community who are informed about research and motivated to bridge the gap between the community and the research establishment)
Funding	Establish a self-sustaining model; identify partners and sponsors; provide services to research sites and activist organizations; apply for grants from government, NGO, and industry groups

PARTAKE, Public Awareness of Research for Therapeutic Advancements through Knowledge and Empowerment; NGO, non-governmental organization
